# TGFβ-1 and TGFBR2 polymorphisms, cooking oil fume exposure and risk of lung adenocarcinoma in Chinese nonsmoking females: a case control study

**DOI:** 10.1186/s12881-015-0170-5

**Published:** 2015-04-10

**Authors:** Yangwu Ren, Zhihua Yin, Kun Li, Yan Wan, Xuelian Li, Wei Wu, Peng Guan, Baosen Zhou

**Affiliations:** Department of Epidemiology, School of Public Health, China Medical University, No. 77 Puhe Road, Shenyang North New Area, Shenyang, Liaoning People’s Republic of China; Liaoning Provincial Department of Education, Key Laboratory of Cancer Etiologic and Prevention (China Medical University), Liaoning, 110122 China

**Keywords:** Transforming growth factor-β, Lung adenocarcinoma, Polymorphisms, Nonsmoking females

## Abstract

**Background:**

Transforming growth factor-β (TGF-β) plays an important role in regulating cellular functions, and many studies have demonstrated important roles for TGF-β in various cancers. Single nucleotide polymorphisms (SNPs) of TGF-β may influence lung carcinogenesis. The aim of this study was to test whether TGF-β1 C509T and TGF-β receptor II (TGFBR2) G-875A polymorphisms were associated with lung adenocarcinoma in nonsmoking females.

**Methods:**

A hospital-based case–control study was performed in Chinese nonsmoking females. Genotyping was performed using TaqMan SNP genotyping assay, and demographic data and environmental exposure were collected by trained interviewers after informed consents were obtained.

**Results:**

A total of 272 (95.4%) cases and 313 (99.4%) controls were successfully genotyped, and the results showed that the polymorphic allele frequencies of C509T and G875A were similar among lung adenocarcinoma patients and controls (*P*=0.589 and 0.643, respectively). However, when the data were stratified for cooking oil fume exposure, the TT genotype of the TGFB1 C509T polymorphism showed a significantly decreased risk for lung adenocarcinoma compared with the CC genotype (adjusted *OR*=0.362, 95% CI=0.149–0.878, *P*=0.025).

**Conclusions:**

TGF-β1 gene C509T polymorphism might be associated with decreased risk of lung adenocarcinoma in Chinese females exposed to cooking oil fumes, but no association was observed TGFBR2 gene G875A polymorphism.

## Background

Lung cancer is one of the leading causes of cancer related death in males and females [1]. Smoking is a major risk factor in lung cancer development, and is responsible for approximately 80% of all lung cancers [2]. However, smoking alone cannot fully explain the epidemiologic characteristics of lung cancer. Epidemiological evidence has shown that only 20% smokers develop lung cancer [3]. In addition, unlike smokers common with squamous cell carcinomas, nonsmokers especially females were more likely to have adenocarcinoma [4]. Furthermore, the incidence and death rate of lung cancer is now declining in men, while continuing to increase in women. Thus, for the nonsmoking female population, it is more important to study the impact factors of lung adenocarcinoma.

Transforming growth factor-β (TGF-β) proteins represent an important family of cytokines that play an important role in regulating cellular functions such as cell proliferation, differentiation, apoptosis, adhesion, invasion and immune response [5-8]. Among the three TGF-β isoforms expressed in mammals, including TGF-β1, TGF-β2 and TGF-β3, TGF-β1 is the most commonly expressed and abundant form in carcinogenesis [6]. TGF-β1 exerts its effects by binding to TGF-β receptor II (TGFBR2) and is associated with the activation of TGF-β receptor I (TGFBR1) and downstream signaling [6,8]. Accumulating evidence has demonstrated that TGF-β signaling plays a dual role in carcinogenesis. TGF-β acts as a tumor suppressor by inhibiting cell proliferation or promoting cell differentiation and apoptosis in the early stage of cancer, and as a tumor promoter by promoting tumor cell invasion, dissemination and immune evasion in advanced stages [5-8].

Several genetic variants in TGF-β1 and TGF-β2 genes and receptors have been identified. The most studied TGF-β1 genetic variant is the C509T polymorphism, which was reported to increase the plasma level of TGF-β1 by influencing its transcriptional activation [9]. Recent studies have identified a relationship between this polymorphism and the risk of cancer in prostate cancer [10], esophageal cancer [11], breast cancer [12], gastric cancer [13] and lung cancer [14,15]. However, in lung cancer-focused studies, the results were inconsistent. The common G875A polymorphism in the promoter region of TGFBR2 is also associated with transcriptional activation of the gene [16]. To the best of our knowledge, there has been no report on the TGFBR2 G875A polymorphism in lung adenocarcinoma.

Our previous study showed that cooking oil fume exposure was a risk factor for lung adenocarcinoma in Chinese nonsmoking females [17,18]. Furthermore, cooking oil fume increased TGF-β1 secretion in lung epithelial cells [19]. Therefore, we speculated that cooking oil fume exposure may show an interaction with TGF-β1 in lung adenocarcinoma development. To test whether the two polymorphisms in TGF-β1 and TGFBR2 were associated with lung adenocarcinoma, a hospital-based case–control study was performed in Chinese nonsmoking females.

## Methods

### Study subjects and data collection

Cases and controls for this study have been described previously [17]. Briefly, we identified newly diagnosed nonsmoking female patients with histologically confirmed lung adenocarcinoma from January 2002 to November 2007. Controls were selected from cancer-free female patients with other lung diseases such as bronchitis, pneumonias, fibrosis, sarcoidosis, and chronic obstructive pulmonary disease. Controls were frequency matched to cases on age (±5 years) and none were smokers. All subjects were all unrelated Han Chinese. This investigation was approved by the Institutional Review Board of China Medical University. After informed consent was obtained from each, cases and controls were interviewed in person to collect demographic data and environmental exposure information by trained interviewers, including demographic characteristics, passive smoking, cooking oil fume exposure, fuel smoke exposure, family history of cancer, occupational exposure and dietary habits. Individuals who had smoked less than 100 cigarettes in their lifetime were defined as nonsmokers. Participants who engaged in frying or deep-frying in the kitchen more than two times each week with reported eye or throat irritation were defined with cooking oil exposure.

### DNA isolation and genotyping

Genomic DNA was extracted from peripheral blood using Proteinase K digestion and standard phenol-chloroform methods. Genotyping was performed on the Applied Bio-systems 7500 FAST Real-Time PCR System (Foster City, CA, USA) using TaqMan single nucleotide polymorphism (SNP) genotyping assay in a 10 μL total reaction volume containing 5 μL 2× TaqMan Genotyping Master mix (Affymetrix Inc., Cleveland, Ohio, USA), 0.5 μL primers and probes (Applied Biosystems, assay ID: C_8708473_10 and C_27491740_10) and 2 μL DNA (15–25 ng/μL). After an initial denaturation at 95°C for 10 min, DNA was amplified by 47 cycles of 92°C for 30 s, and 62°C for 1 min. Ten percent of all samples were randomly selected and genotyped again for quality control.

### Statistical analysis

Statistical analyses were performed using SPSS version 16.0 software (SPSS Inc., Chicago, IL). A value of *P* < 0.05 was considered statistically significant. Deviations from the Hardy–Weinberg equilibrium for each SNP were assessed using the Pearson *χ*^2^ test. The characteristics of the patients with lung adenocarcinoma and the controls were compared using Pearson *χ*^2^ test. The odds ratio (*OR*) and its 95% confidence interval (*CI*) with adjustment for age were obtained by logistic regression method to determine the correlation between the two polymorphisms, exposure information and the risk of lung adenocarcinoma.

## Results

Genomic DNA samples were extracted from 285 lung adenocarcinoma cases and 315 cancer-free controls. A total of 63 samples (31 cases and 32 controls) were randomly selected for genotyping replication, and the concordance rate was 100%. A final 272 cases and 313 controls were successfully genotyped and included for further analyses. The demographic variables and environmental risk factors of the patients are listed in the Table 1. All cases were lung adenocarcinoma patients and females. There were no significant differences between the cases and controls in the distribution of age, income, education, family history of cancer, passive smoking and fuel smoke exposure. However, more cooking oil fume exposure was reported in the cases group (crude-*OR*=1.554, 95% *CI*=1.092–2.221, *P*=0.013).

**Table 1:** **Selected variable in cases and controls**

**Variable**	**Cases n (%)**	**Controls n (%)**	***P***
Sample size	272	313	
Age (years)	54.0±12.1	54.2±9.1	0.825
Income (yuan/mouth)	622.4±377.2	561.0±388.1	0.073
Education			0.945
Never	24	27	
Elementary school	129	156	
Junior school	79	85	
Senior school and upwards	40	45	
Family history of cancer	35	33	
Passive smoking	161(59.2%)	178(56.9%)	0.570
Fuel smoke exposure	78(28.7%)	85(27.2%)	0.683
Cooking oil fume exposure	98(36.0%)	83(26.5%)	0.013

The genotype and polymorphic allele frequencies of TGF-β1 C509T and TGFBR2 G875A polymorphisms of the cases and controls are shown in Table 2. The genotype distributions of both polymorphisms among the controls were in Hardy–Weinberg equilibrium (*P*=0.528 and 0.361 for TGF-β1 C509T and TGFBR2 G875A, respectively). No differences of genotypes for TGF-β1 C509T and TGFBR2 G875A between the cases and the controls were detected (*P*=0.589 and 0.643, respectively) or polymorphic allele frequencies (*P*=0.451 and 0.731, respectively). However, when we performed the combined analyses according to the number of variant loci for these two SNPs (Table 2), those with one variant locus had a decreased risk of lung adenocarcinoma. However, this was not the case for carriers of two variant loci (age-adjusted *OR*=0.591, 95% *CI*=0.374–0.935, *P*=0.025 for subjects carrying one variant locus; age-adjusted *OR*=0.675, 95% *CI*=0.399–1.141, *P*=0.142 for subjects carrying two variant loci).

**Table 2:** **TGFB1 C-509T and TGFBR2 G-875A polymorphisms and lung adenocarcinoma risk**

**Polymorphism**	**Cases**	**Controls**	**OR**_**adg**_	**95% ** ***CI***	***P***
**TGF-β1 C-509T**					
CC	78(28.7)	79(25.2)	1.0		
CT	138(50.7)	162(51.8)	0.847	0.574-1.249	0.402
TT	56(20.6)	72(23.0)	0.768	0.480-1.228	0.270
CT/TT	194(71.3)	234(74.8)	0.822	0.569-1.188	0.298
C	294(54.0)	320(51.1)	1.0		
T	250(46.0)	306(48.9)	0.878	0.697-1.106	0.270
**TGFBR2 G-875A**					
GG	182(66.9)	202(64.5)	1.0		
GA	80(29.4)	102(32.6)	0.865	0.605-1.237	0.427
AA	10(3.7)	9(2.9)	1.223	0.486-3.078	0.669
GA/AA	90(33.1)	111(35.5)	0.895	0.634-1.263	0.528
G	444(81.6)	506(80.8)	1.0		
A	100(18.4)	120(19.2)	0.946	0.704-1.271	0.711
Combined analysis					
0 Variant locus	54(19.9)	42(13.4)	1.0		
1Variant locus	152(55.9)	197(62.9)	0.591	0.374-0.935	0.025
2 Variant locus	66(24.3)	74(23.6)	0.675	0.399-1.141	0.142

*Abbreviation:* *OR*_*adg*_ age-adjusted odds ratio, *CI* confidence interval.

In analysis stratified by cooking oil fume exposure, no association was found between the TGF-β1 C509T polymorphism and the risk of lung adenocarcinoma in subjects without exposure to cooking oil fumes (Table 3). However, individuals exposed to cooking oil fumes with TT genotype were at a significantly decreased risk of lung adenocarcinoma compared with those with the CC genotype (age-adjusted *OR*=0.362, 95% *CI*=0.149–0.878, *P*=0.025) and in a dose–response manner (*χ*^2^=4.558, *P*=0.033). Regarding the TGFBR2 G875A polymorphism, no difference of genotype distribution between the two groups was detected. In stratified analyses by fuel smoke exposure and passive smoking status, as shown in Table 4 and Table 5, a nonsignificant association between the two polymorphisms and lung adenocarcinoma was observed. To evaluate the interaction of genetic polymorphism with cooking oil fume exposure in lung adenocarcinoma, a logistic regression was performed; the crude *OR* and age-adjusted *OR* value for the TT gene type with cooking oil fume exposure were 0.331 (95% *CI*=0.116–0.942, *P*=0.038) and 0.343 (95% *CI*=0.12–0.976, *P*=0.045), respectively (data not shown). The *P* values for the interaction of TGF-β1 and TGFBR2 with cooking oil fume exposure were 0.046 and 0.005, respectively.

**Table 3:** **TGF-β1 C-509T and TGFBR2 G-875A polymorphisms and lung adenocarcinoma risk, stratified by cooking oil fume exposure**

**Polymorphism**	**Exposure to cooking oil fume**					**Non exposure to cooking oil fume**					***P***_**interaction**_
	**Cases**	**Controls**	**OR**_**adg**_	**95% ** ***CI***	***P***	**Cases**	**Controls**	**OR**_**adg**_	**95% ** ***CI***	***P***	
**TGF-β1 C-509T**											0.046
CC	31	20	1.0			47	59	1.0			
CT	54	40	0.887	0.441-1.784	0.737	84	122	0.841	0.523-1.354	0.477	
TT	13	23	0.362	0.149-0.878	0.025	43	49	1.065	0.606-1.870	0.827	
CT/TT	67	63	0.693	0.358-1.342	0.277	127	171	0.906	0.578-1.420	0.666	
**TGFBR2 G-875A**											0.005
GG	59	60	1.0			123	142	1.0			
GA	34	23	1.451	0.761-2.766	0.258	46	79	0.674	0.435-1.047	0.079	
AA	5	0				5	9	0.629	0.205-1.929	0.417	
GA/AA	39	23	1.664	0.883-3.136	0.115	51	88	0.670	0.439-1.022	0.063	

*Abbreviation:* *OR*_*adg*_ age-adjusted odds ratio, *CI* confidence interval.

**Table 4:** **TGF-β1 C-509T and TGFBR2 G-875A polymorphisms and lung adenocarcinoma risk, stratified by fuel smoke exposure**

**Polymorphism**	**Exposure to fuel smoke**					**Non exposure to fuel smoke**					***P***_**interaction**_
	**Cases**	**Controls**	**OR**_**adg**_	**95% ** ***CI***	***P***	**Cases**	**Controls**	**OR**_**adg**_	**95% ** ***CI***	***P***	
**TGF-β1 C-509T**											0.685
CC	21	17	1.0			57	62	1.0			
CT	43	47	0.706	0.325-1.533	0.378	95	115	0.886	0.564-1.394	0.601	
TT	14	21	0.501	0.195-1.289	0.152	42	51	0.883	0.512-1.523	0.601	
CT/TT	57	68	0.642	0.305-1.353	0.244	137	166	0.885	0.578-1.356	0.576	
**TGFBR2 G-875A**											0.922
GG	61	57	1.0			121	145	1.0			
GA	15	24	0.594	0.280-1.257	0.173	65	78	0.987	0.655-1.488	0.951	
AA	2	4	0.465	0.082-2.648	0.388	8	5	1.901	0.606-5.963	0.271	
GA/AA	17	28	0.574	0.282-1.167	0.125	73	83	1.043	0.700-1.553	0.837	

*Abbreviation:* *OR*_*adg*_ age-adjusted odds ratio, *CI* confidence interval.

**Table 5:** **TGF-β1 C-509T and TGFBR2 G-875A polymorphisms and lung adenocarcinoma risk, stratified by passive smoking**

**Polymorphism**	**Exposure to passive smoking**					**Non exposure to passive smoking**					***P***_**interaction**_
	**Cases**	**Controls**	**OR**_**adg**_	**95% ** ***CI***	***P***	**Cases**	**Controls**	**OR**_**adg**_	**95% ** ***CI***	***P***	
**TGF-β1 C-509T**											0.648
CC	21	17	1.0			57	62	1.0			
CT	43	47	0.706	0.325-1.533	0.378	95	115	0.886	0.564-1.394	0.601	
TT	14	21	0.501	0.195-1.289	0.152	42	51	0.883	0.512-1.523	0.601	
CT/TT	57	68	0.642	0.305-1.353	0.244	137	166	0.885	0.578-1.356	0.576	
**TGFBR2 G-875A**											0.213
GG	61	57	1.0			121	145	1.0			
GA	15	24	0.594	0.280-1.257	0.173	65	78	0.987	0.655-1.488	0.951	
AA	2	4	0.465	0.082-2.648	0.388	8	5	1.901	0.606-5.963	0.271	
GA/AA	17	28	0.574	0.282-1.167	0.125	73	83	1.043	0.700-1.553	0.837	

*Abbreviation:* *OR*_*adg*_ age-adjusted odds ratio, *CI* confidence interval.

For multiple comparisons, a Bonferroni correction was performed. We genotyped 11 SNPs on these subjects, involving 106 subsequent analyses in a total of five studies, including four previously published studies [17,18,20,21] and the current study. The adjusted statistically significant level was 4.72 × 10^−4^, and therefore all the associations between polymorphisms and lung adenocarcinoma were not statistically significant in the study.

## Discussion

Lung cancer is currently the most common malignancy in the world, and adenocarcinoma is most common in females, especially in those who have never smoked. To explore mechanisms involved in lung adenocarcinoma, we investigated the influence of the TGF-β1 and TGFBR2 polymorphisms on the risk of lung adenocarcinoma in a population-based case–control study. The TGF-β1 C509T polymorphism was significantly associated with the risk of lung adenocarcinoma in individuals exposed to cooking oil fumes, and protective effects were observed in a dose–response manner. This finding suggests that the TGF-β1 C509T polymorphism could be used as genetic susceptibility marker for early diagnosis. The 5-year survival rate of lung cancer patients was only 16%, but the survival rate of clinical stage I could reach 59% [22]. This indicates that early diagnosis may increase survival and improve the quality of life for lung cancer patients.

In the current study, the frequencies of the C509T and G875A alleles among the healthy controls were 0.489 and 0.192, respectively, similar to previous studies in a Chinese population [9,11,13,23]. Although the frequencies of CT and TT homozygotes among lung adenocarcinoma patients were lower than those in the control group (50.7% vs. 51.8% in CT, 20.6% vs. 23.0% in TT, respectively), no significant association between the TGF-β1 C509T polymorphism and the risk of lung cancer was found. This result was different from the study of Kang *et al.* [14], but similar to the findings of Park *et al.* [15]. Several studies showed that TGF-β1 C509T polymorphisms are associated with TGF-β1 production [9,14,24-26]. Grainger *et al.* [24] reported that the variant T allele at C509T was associated with higher plasma levels, and the TT homozygote was associated with highest plasma levels. Luedecking *et al.* [26] reported that the variant T allele was associated with increased transcriptional activity. However, identical conclusions were reported by Wang *et al.* [9] and Peng *et al.* [23], who found that the C allele rather than T allele was associated with higher plasma levels and transcriptional activity, respectively. Differences in ethnic background and genetic heterogeneity in different cancers may be the main reason for these different results.

TGFBR2 is a key receptor in regulating the TGF-β signaling pathway by binding to TGF-β and activating TGFBR1, so mutations in TGFBR2 may inactivate or activate the TGF-β signaling pathway [5,6]. To date, many mutations in the TGFBR2 promoter have been reported [27,28] that may contribute to reduced receptor expression in tumors. However, the G875A polymorphism is the only one in which minimum allele frequency was less than 0.05 in the promoter region of TGFBR2 [16], and this polymorphism was reported as associated with transcription activities of the gene [16]. To the best of our knowledge, this is the first study that focused on the relationship between TGFBR2 G875A polymorphism and lung adenocarcinoma risk. However, no association was found. Similar studies in other cancers such as gastric cancer [13] and esophageal squamous cell carcinoma [11] in Chinese populations reported different results, probably because of genetic heterogeneity in the pathogenesis of different cancers.

A previously published genome-wide association study (GWAS) on the risk of developing lung cancer among women in Asia observed no evidence of association for TGF-β1 and TGFBR2 [29]. Our results are consistent with the GWAS findings. Additionally, in our analysis stratified by cooking oil fume exposure, a significant association between the TGF-β1 C509T polymorphism and the risk of lung adenocarcinoma in subjects with cooking oil fume exposure was found. The published GWAS has stronger evidence and extrapolation, and the present study could provide some clues for lung cancer in the population living in the northeastern part of China. Some *in vitro* results support the results of our study. Previous studies have shown that exposure to cooking oil fume condensates could inhibit cell growth, increase TGF-β1 secretion and induce oxidative stress in human lung epithelial cells [19], suggesting that TGF-β1 may play a role in the adverse health effects of oil fumes. However, the exact molecular mechanisms are far from understood. Reactive oxygen species can lead to increased TGF-β1 release in human lung epithelial cells [30], and Leonarduzzi *et al.* [31] reported that 4-hydroxy 2,3-nonenal, a major aldehyde end product of lipid peroxidation, can also induce TGF-β1 secretion in macrophages. This indicates that TGF-β1 may play an important role in oxidative stress induced by cooking oil fumes. In addition, benzo[a]pyrene (B[a]P), a product of heated rapeseed oil, has been also demonstrated to increase TGF-β1 mRNA levels [32]. B[a]P can lead to cell carcinogenesis by causing DNA damage [33], therefore we speculate that TGF-β1 might act as an protector by regulating cell proliferation and apoptosis.

This study includes several limitations. First, the sample size might not be large enough to detect the low penetrance effect of the genes, especially for stratified analysis. However, we used a trend *χ*^2^ test to explore the dose–response manner of lung adenocarcinoma risk and TGF-β1 C509T polymorphism in nonsmoking female exposure to cooking oil fumes, and the power of this result is 0.957 (data not shown). Second, the rate of fuel smoke exposure is 37.2%, which is higher than that of other regions of China, and we did not find any association between fuel smoke and lung adenocarcinoma. We matched the controls to the cases on age and sex, and age-adjusted ORs were calculated by logistic regression to decrease potential confounding bias caused by age. Finally, hospital-based studies are likely to include some selection bias and information bias. The exposure assessment of cooking oil fume and fuel smoke was performed by subjective reports, which has limited accuracy. Further study is needed to assess these exposures using controlled measurements and instruments.

## Conclusions

Our study demonstrates that the variant genotypes in the promoter of TGF-β1 might decrease the risk of lung adenocarcinoma in females exposed to cooking oil fumes (TT vs. CC: *OR*=0.362, *P*=0.025), but no effect was observed with the TGFBR2 G875A polymorphism. However, because genetic polymorphisms often vary in different ethnic groups, these findings need to be confirmed in larger groups. Further studies also need to clarify the exact molecular mechanisms of interaction between cooking oil fumes and TGF-β1.

## Abbreviations

SNPs: Single nucleotide polymorphisms
TGFB1: Transforming growth factor-β1
TGFBR2: Transforming growth factor receptorII
OR: Odds ratio
CI: Confidence interval
B[a]P: Benzo[a]pyrene
